# SLEEP QUALITY AND DURATION AND RISK OF FALLS IN OLDER ADULT WOMEN: THE STUDY OF WOMEN’S HEALTH ACROSS THE NATION (SWAN)

**DOI:** 10.1093/geroni/igae098.0360

**Published:** 2024-12-31

**Authors:** Jillian Baker, Michelle Hood, Leslie Swanson, Christopher Kline, Kelly Ylitalo, Jane Cauley, Dr Robin Green, Carrie Karvonen-Gutierrez

**Affiliations:** University of Michigan School of Public Health, Ann Arbor, Michigan, United States; University of Michigan, Ann Arbor, Michigan, United States; University of Michigan, Ann Arbor, Michigan, United States; University of Pittsburgh, Pittsburgh, Pennsylvania, United States; Baylor University, Waco, Texas, United States; University of Pittsburgh School of Public Health, Pittsburgh, Pennsylvania, United States; Einstein College of Medicine, Bronx, New York, United States; University of Michigan, Ann Arbor, Michigan, United States

## Abstract

**Background:**

As the leading cause of injury in American older adults, falls can be consequential for function and mortality, but are preventable. Sleep may be a modifiable risk factor for falls. We hypothesized poorer sleep quality and shorter sleep duration would be associated with increased risk of falls and a greater number of falls.

**Methods:**

The Study of Women’s Health Across the Nation is a multi-ethnic, community-based longitudinal study of women followed through and beyond the menopausal transition. In 2010-2011, participants self-reported measures of sleep quality and duration. In 2015-2016, participants reported number of falls in the year prior. Adjusted models included demographic and socioeconomic factors, and health conditions.

**Results:**

Women who reported frequent (5+ times/week) trouble falling asleep and frequent waking in the middle of the night in 2010-2011 had a 27% (RR=1.27, 95% CI=1.01,1.62) and 24% (RR=1.24, 95% CI=1.01,1.48) increased risk of a subsequent fall. Frequent trouble falling asleep and short sleep duration (< 6 hours) were both associated with higher likelihood of recurrent falls (>=3 falls vs. <=1 fall) (OR=2.54, 95% CI=1.30,4.96; OR=1.80, 95% CI=1.07,3.05). Conclusions: Multiple indicators of poor sleep including trouble falling asleep, frequent waking, and short duration were associated with increased fall burden in older adult women. As falls pose a major risk factor for decline in health and mortality, promoting adequate, high-quality sleep may be an essential component in fall prevention.

